# A Fluid–Structure Interaction Computational Study of Residual Aortic Dissection to Investigate the Influence of Mechanical Behaviour of Wall and Flap on Flows

**DOI:** 10.1002/cnm.70120

**Published:** 2025-12-04

**Authors:** C. Guivier‐Curien, V. Deplano

**Affiliations:** ^1^ IRPHE UMR7342 Aix Marseille Université, CNRS, Centrale Méditerranée Marseille France

**Keywords:** computational modelling, flow resistance, mechanical behaviour, thoracic aortic dissection, wall and flap motion

## Abstract

Residual thoracic aortic dissection (RTAD) is a pathology whose patient‐dependent evolution is an important clinical issue, and for which numerical fluid–structure interaction (FSI) models can be helpful. However, the proposal of an ad hoc mechanical wall and flap model remains a challenge. To make progress on this issue, we seek to understand the respective influence of the flap and the wall in interaction with the flow in RTAD. Based on a patient's RTAD geometry, we established different FSI models to simulate the mechanical behaviour of the wall and flap. We varied the Young's modulus of the wall between 1.2 MPa (W12) and 2.7 MPa (W27) and the Young's modulus of the flap between 0.6 MPa (F06) and 1.2 MPa (F12), resulting in 3 different cases to study (F06W12, F12W12 and F06W27) and allowing a relative comparison. Structural displacements and stresses are equivalent in F06W12 and F12W12, resulting in equivalent flow characteristics. When comparing F06W12 with F06W27, we show that a stiffer wall reduces flap motion by 49.8%, 51% and 52% respectively, around the first entry tear, second and third one respectively. The difference in flow pressure between channels, which reflects the resistance to flow, is very small (about 1–2 mmHg) and similar for all 3 cases. This result seems to be highly related to the current geometry with one entry and two re‐entry tears. Our results show that the wall is the main driver of the overall mechanical behaviour of the RTAD. We demonstrated that a stiffer pathological wall leads to smaller flap displacements, which is consistent with clinical observations in the chronic phase.

## Graphical abstract

The wall is the main driver of the overall mechanical behaviour of the residual thoracic aortic dissection. During the chronic phase, flap motion is less important; the main focus should be on flow patterns, to quantify flow resistance and lumen patency.

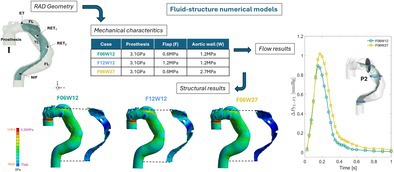

## 1 Introduction

Thoracic aortic dissection (TAD) is one of the most life‐threatening cardiovascular diseases resulting from the occurrence of a tear in the arterial wall allowing blood to pass through and split longitudinally the wall. A resulting false lumen appears opposite the true one, and the two lumens are separated by a so‐called intimal flap. From a mechanical point of view, the intimal flap is a deformable membrane in a compliant tube in which a pulsatile fluid flows. Understanding the interactions between these structures and the hemodynamic factors could provide new insights into the problems clinicians face daily, such as decision support for intervention or re‐intervention to prevent adverse events.

Numerical modelling is of interest to study TAD and help the clinicians make therapeutic choices. Biomechanical interactions between blood and both aortic wall and intimal flap can be studied through fluid–structure interaction (FSI) models which are the reference for capturing the complexity of the pathology. TADs that are observed in the descending aortic segment were the subject of few FSI studies [1, 2, 3]. Modelling TAD in the descending aortic segment persisting after previous surgery on the ascending part (called residual TAD—RTAD) is also scarce [4, 5, 6, 7] whereas the clinical issue is important because in more than 45% of RTAD cases, clinical adverse events occur leading to new rapid reintervention [8].

However, to be relevant, such FSI study has to consider not only the specific geometry of the patient, but also the mechanical behaviour of the tissues involved. Medical imaging, such as computed tomography angiography (CTA), provides access to patient‐specific geometries with a millimetre resolution, and is now widely and almost systematically implemented in Computational Fluid Dynamics (CFD) and FSI models of the aorta [9, 10]. In some cases, boundary conditions and more especially 3D velocity inlet conditions can also be accessed through imaging such as phase contrast Magnetic Resonance Imaging (PC‐MRI) [11, 12] or 4D flow Magnetic Resonance Imaging (4D‐MRI) [13]. On the contrary, the local patient‐specific mechanical behaviour of aortic walls is not currently accessible with any of these imaging modalities. The mechanical structural modelling choice is thus generally a limitation when dealing with patient‐specific FSI studies. To overcome this lock, very few in vitro data are available in the literature concerning (R)TAD, mainly because samples are very scarce to be experimentally characterised. Consequently, the same mechanical characteristics on the wall and the flap are often considered, even though they are not mechanically identical. Moreover, in a previous work [7], we have shown that a FSI study, including both compliant flap and wall, and not only flap or wall, is of importance to catch the mechanical behaviour complexity of the pathology. Taking into account the mechanical behaviour of the flap but rigid wall leads to similar results to those obtained in a rigid model concerning the dynamics of flow. Indeed, flap motion is reduced to about 0.25 mm when this motion is 4‐ to 5‐fold higher when FSI is concerned.

To our knowledge, only three FSI studies with patient‐specific geometry consider flap and wall as mechanically not equivalent. In the same patient geometrical model, Zhu et al. [5] varied the Young's modulus of the wall while keeping the same Young's modulus of the flap. Conversely, Baumler et al. [4] varied the flap Young's modulus but kept the same Young's modulus of the wall. In a recent FSI study from the same group, Schussnig et al. [6] investigated a complex structural model with layer‐specific anisotropic properties of the aortic wall. They compared three models with different flap stiffnesses but also different overall mechanical behaviour (hyperelastic and elastic model). All these studies reported a very wide range of intimal flap displacement, from 1 to 2 mm in Zhu et al. [5], 1.7–3.5 mm in Schussnig et al. [6], and up to 10 mm in Baumler et al. [4] when using a very compliant flap and a wall 40 times stiffer. No study has examined the respective influence of flap and wall on interactions with blood when their respective Young's modulus varies in the same geometry.

All these observations lead us to question the relevance of the mechanical choices that are made concerning flap and wall mechanical behaviour when dealing with a computational model regarding the understanding of the pathology. These mechanical choices need to be addressed to assess their influence on the description of complex flow and structural patterns that may be involved in the evolution of pathology up to the triggering of an adverse clinical event. In a previous study, we compared rigid modelling with an FSI case in which flap and wall had the same mechanical behaviour [7]. To gain new insights in the FSI computational model regarding the clinical question and to better understand the respective mechanical role of the wall and flap and their interactions with flows in RTAD, we focus in the present study on different mechanical structural behaviours from the same RTAD geometry, by varying both flap and wall Young's modulus. We question the relevance of these choices in terms of the evolution of the pathology from acute to chronic phase.

## 2 Materials and Methods

A 68‐year‐old man was admitted to the Hôpital de la Timone (Marseille, France) with a type A dissection surgically treated with a prosthesis on the ascending thoracic aortic segment. A residual dissection persisted in the descending part. The patient gave his informed consent. From CTA images (1 mm thickness slice, plane resolution 0.89 mm/px, 512 × 512 pixels, 16‐bit grayscale images), the patient's thoracic aorta geometry was reconstructed from the aortic root to the celiac trunk with Invesalius 3.1 software (CTI, Brazil). The 3 aortic branches from the aortic arch were included. The false lumen (FL) and true lumen (TL) were segmented in the descending aorta, separated by the flap of varying thickness, depending on the position and as a result of the segmentation (mean value = 1.85 mm). A main entry tear (ET) and 2 other re‐entry tears (RET_1_, RET_2_) of equivalent sizes (391 mm^2^, 424 mm^2^ and 396 mm^2^ for ET, RET_1_ and RET_2_, respectively) were identified and assumed to be elliptical with the help of the clinician who validated all the steps leading to the geometry obtained. The image contrast was not sufficient to correctly obtain the thickness of the aortic wall. Therefore, according to Tang et al. [14], the aortic wall thickness was assumed to be constant with a value of 2.5 mm. The prosthesis on the ascending part with a *D* = 40 mm diameter was supposed to be cylindrical and with a thickness equivalent to the aortic wall.

### 2.1 Numerical Modelling

The model was composed of a fluid domain and a solid domain including a flap, aortic wall and prosthesis.

### 2.2 Structural Modelling

All structural parts were supposed to be linear elastic and isotropic materials. Table 1 shows the different cases that were investigated in the present study: the Young's modulus of the prosthesis on the ascending part corresponded to the manufacturer data; the Young's modulus of the flap (*F*) and the wall (*W*) were changed according to the studied case. Values were chosen in accordance with echocardiographic measurements [15] on the ascending aorta in which the modulus of Peterson had been assessed to lead to a Young's modulus for the healthy wall of about 1.2 MPa and was in agreement with values found in the literature [5]. A stiffer value of 2.7 MPa corresponded to the increased stiffness observed in patients [16, 17].

**TABLE 1:** Different cases and the corresponding Young's modulus value for wall (*W*) and flap (*F*).

Case	Prosthesis (GPa)	Flap (*F*, MPa)	Aortic wall (*W*, MPa)	Ratio (*W*/*F*)
*F06W12*	3.1	0.6	1.2	2
*F06W27*	3.1	0.6	2.7	4.5
*F12W12*	3.1	1.2	1.2	1

Boundary condition was embedded condition for the aortic root and only radial displacements were allowed for other boundaries (3 aortic arch outlets and descending aortic outlet). Lateral walls were free to move. To model the state of physiological pre‐stress, a pressure condition of 80 mmHg was imposed on all internal walls of the solid domain. The resulting constraints were applied to start the simulation with zero displacements. The solid domain was discretised in 72,203 elements for the flap, 436,217 elements for the aortic wall and, 33,295 elements for the prosthesis. Special care had been taken to ensure the conformity of the meshes between the different structural parts.

Large deformations were allowed, and Rayleigh damping was turned on with *α* = 50 and *β* = 0.1 to improve the convergence of FSI simulations [5].

### 2.3 Fluid Modelling

The unsteady and incompressible flow was assumed to be laminar and the fluid behaved as a shear‐thinning one using the Carreau–Yasuda model with constants from Leuprecht et al. [18].

$$\frac{\mu -\mu_{\infty }}{\mu_{0}-\mu_{\infty }}={\left[1+{\left(\lambda \dot{\gamma }\right)}^{a}\right]}^{\left(n-1\right)/a}$$

with $\mu_{\infty }=0.0035\;\mathrm{Pa}\;s$, $\mu_{0}=0.16\;\mathrm{Pa}\;s$, λ=8.2s, n=0.2128 and a=0.64.

Fluid boundary condition was a velocity condition (Womersley velocity profiles) derived from a flow rate from Olufsen et al. [19] for the main entrance at the aortic root. Three‐element Windkessel model (3‐EWM) was tuned at each outlet (aortic branches and descending aorta), allowing pressure profiles to be implemented [20]. Parameter set of values used for 3‐EWM (2 resistances and 1 compliance) is presented in Table 2.

**TABLE 2:** Parameter set of values used for the 3‐EWM (*R*_1_ proximal resistance, *R*_2_ resistance and *C* compliance).

	3 aortic arch outlets			Descending outlet
	First branch	Second branch	Third branch	Abdominal aorta
*R*_1_ [10^7^ kg m^−4^ s^−1^]	1.04	1.06	1.12	2.37
*R*_2_ [10^9^ kg m^−4^ s^−1^]	1.39	1.96	1.91	0.15
*C* [10^−9^ kg^−1^ m^4^ s^2^]	3.09	1.82	2.45	9.19

The mean Reynolds number (Remean=ρfvfmeanDμ∞) was equal to 1279 with $\rho_{f}=1060\;\mathrm{kg}\;m^{-3}$ the fluid density and $v_{f\text{mean}}$ the mean entrance velocity; subscript f denotes fluid. The Womersley number $\alpha =R\sqrt{\frac{2{\pi \rho }_{f}}{T\mu_{\infty }}}$ is equal to 27.6, with *T* the cardiac period (1 s).

The fluid domain was discretised in 1,276,255 elements combining tetrahedral ones in the core region and prismatic elements in boundary layers to correctly catch the boundary layer which thickness was found to be 0.725 mm according to Fung's definition.

The pressure–velocity linkage was resolved using the SIMPLE (Semi‐Implicit Method for Pressure‐Linked Equations) iterative solution strategy. The least squares cell‐based algorithm was used for the spatial discretisation of gradients. The pressure was solved using a second‐order scheme, as was the momentum. Finally, a second‐order implicit scheme was employed for the time formulation.

### 2.4 Fluid–Structure Interaction

Equations to solve were:

- For the fluid part (*p* pressure, $\vec{v_{f}}$ velocity, $\tau_{f}$ the viscous stress tensor)
$$\nabla .\vec{v_{f}}=0$$

with $\vec{v_{f}}$ the fluid velocity

$$\rho_{f}\frac{\partial \;\vec{v_{f}}}{\partial t}+\rho_{f}\left(\left(\vec{v_{f}}-\vec{w}\right).\nabla \right)\;\vec{v_{f}}=-\nabla p+\nabla .\tau_{f}$$

- For the solid part ($\vec{u_{s}}$ displacement, $\sigma_{s}$ stress tensor, $\rho_{s}$ solid density, $\vec{F_{s}}$ density of forces acting on the solid)
$$\nabla .\sigma_{s}+\vec{F_{s}}=\rho_{s}\;\frac{\partial^{2}\vec{u_{s}}}{\partial t^{2}}$$

The equations to solved at the interface were the kinematic condition $\vec{v_{f}}=\frac{\partial \;\vec{u_{s}}}{\partial t}$ and the dynamic condition $\sigma_{s}.\vec{n_{s}}=\sigma_{f}.\vec{n_{f}}$, where subscript s denotes solid; $\sigma_{f}$ is the fluid stress tensor, $\vec{n}$ is the boundary normal vector and $\vec{n_{s}}=-\vec{n_{f}}$ at the interface. Arbitrary Lagrangian Eulerian method was used to solve sequentially fluid and solid equations in a 2‐way FSI process. A time step of 0.001 s was imposed and for each, 10 iteration coupling steps were imposed to allow convergence of the solution.

Ansys Workbench R2 2020 (ANSYS Inc., Canonsburg, USA) was used to perform all the simulations. More details on the numerical implementation of the fluid and solid domain, as well as the coupling method can be found in Deplano et al. [7]. Tests were performed on the mesh sensitivity to select the current mesh [20]. Three periods were performed to ensure convergence [7]. The results are from the third one.

## 3 Results

The results are presented for the three cases F06W12, F12W12 and F06W27 (Table 1). Case F06W12 allows comparison with F12W12 to investigate more deeply the role of the mechanical behaviour of the flap whereas a comparison of F06W12 with F06W27 allows direct investigation of the influence of the mechanical behaviour of the wall.

### 3.1 Structural Features

#### 3.1.1 Displacements

**FIGURE 1:**
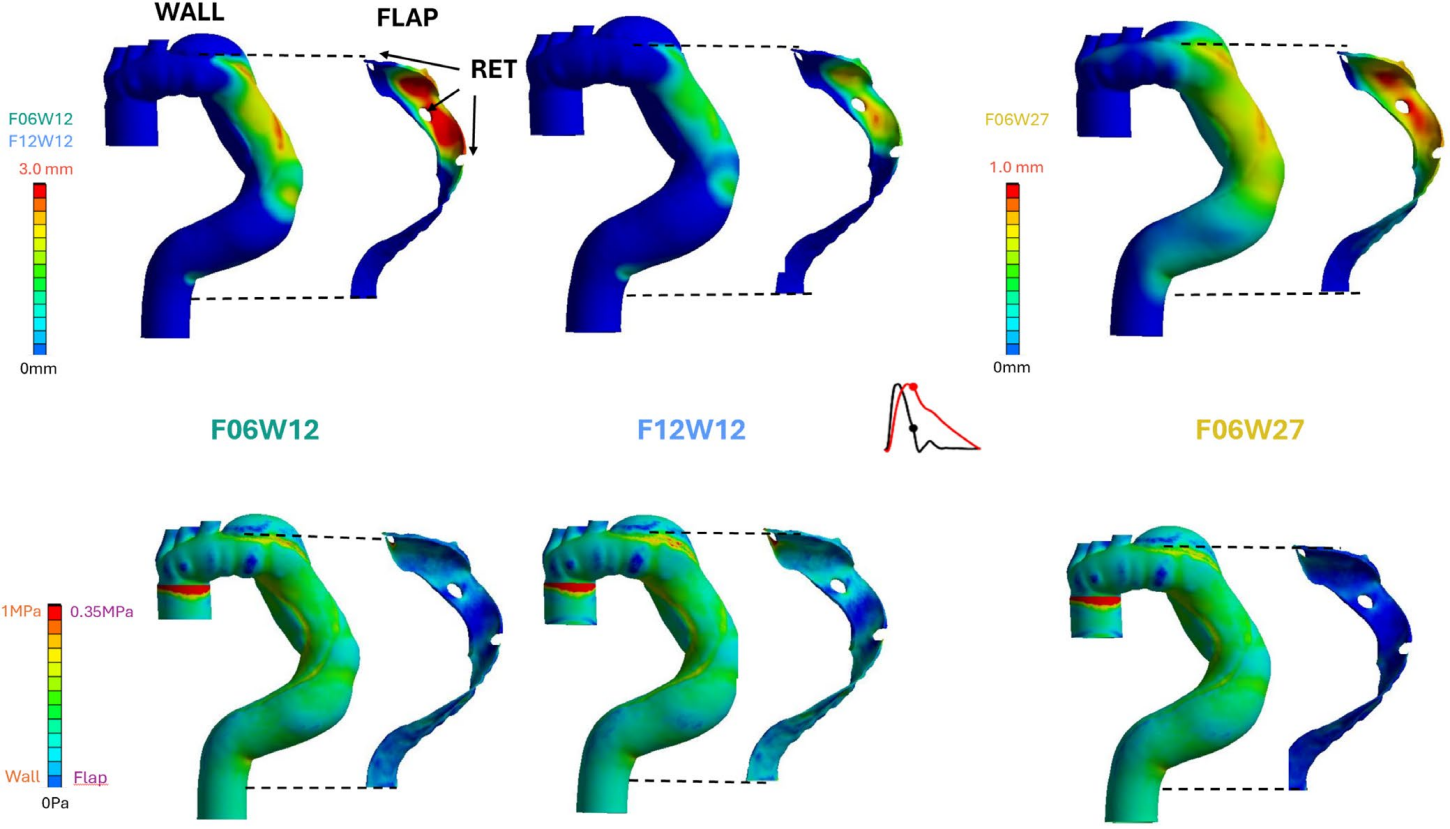
Upper line: Displacement field at the peak of pressure for F06W12 (left), F12W12 (middle) and F06W27 (right), lower line stress field at the peak of pressure for F06W12 (left), F12W12 (middle) and F06W27 (right): RET, re‐entry tear. For each case, arterial wall and flap results are presented, the location of the top and bottom of the flap in the wall is indicated by the black dotted lines. Note that the range of values for displacement field is not the same for F06W27 than for the others.

The displacement values are defined as the displacement magnitude minus the minimum of the displacement magnitude recorded over the period (Figure 1, upper line). Whatever the case, we observe that the displacement patterns on the aortic walls are similar and the displacement curve around the tears had a similar shape. However, the magnitude is different with higher values for F06W12 and F12W12 than for F06W27. From a global point of view, the smallest values are found in the prosthesis zone which is very rigid in comparison to the flap and wall. Higher values are observed on the first part of the descending aorta downstream of the aortic arch for the wall and around tear entry and re‐entries for the flap. In detail, the values recorded in F06W27 are lower than in F06W12 and F12W12: at *t* = 0.36 s (pressure peak) a maximum value of 1.07 mm is recorded on the wall for F06W12 but only 0.66 mm for F06W27. Similarly, at the same instant, on the flap, the average displacement value around RET1 is maximum and equals 1.29 1.29 mm for F06W12, 1.15 mm for F12W12 and 0.66 mm for F06W27. On average over the cardiac period, average values recorded around ET, respectively RET1 and RET2, are 12.8% higher in F06W12 than in F12W12, respectively 12.0% and 13.0%; and 49.8% higher in F06W12 than in F06W27, respectively 51.0% and 52.0%.

#### 3.1.2 Stresses

Stresses are presented as Von Mises stress magnitudes (Figure 1, lower line). A maximum of stress with a value of 1 MPa is recorded in all cases at the junction between the prosthesis and the aortic wall due to the great mismatch in compliance between the parts. On the flap, the average stress value around ET is maximal at *t* = 0.36 s, and reaches a maximum of 142 kPa for F12W12, 87 kPa for F06W12 and 42 kPa for F06W27. On average over the cardiac period, the average stress values recorded around ET, respectively RET1 and RET2, are 63.0% lower in F06W12 than in F12W12, (respectively, 42.7% and 48.2%) and 51.0% higher in F06W12 than in F06W27, (respectively, 46.4% and 52.2%).

### 3.2 Flow Features

**FIGURE 2:**
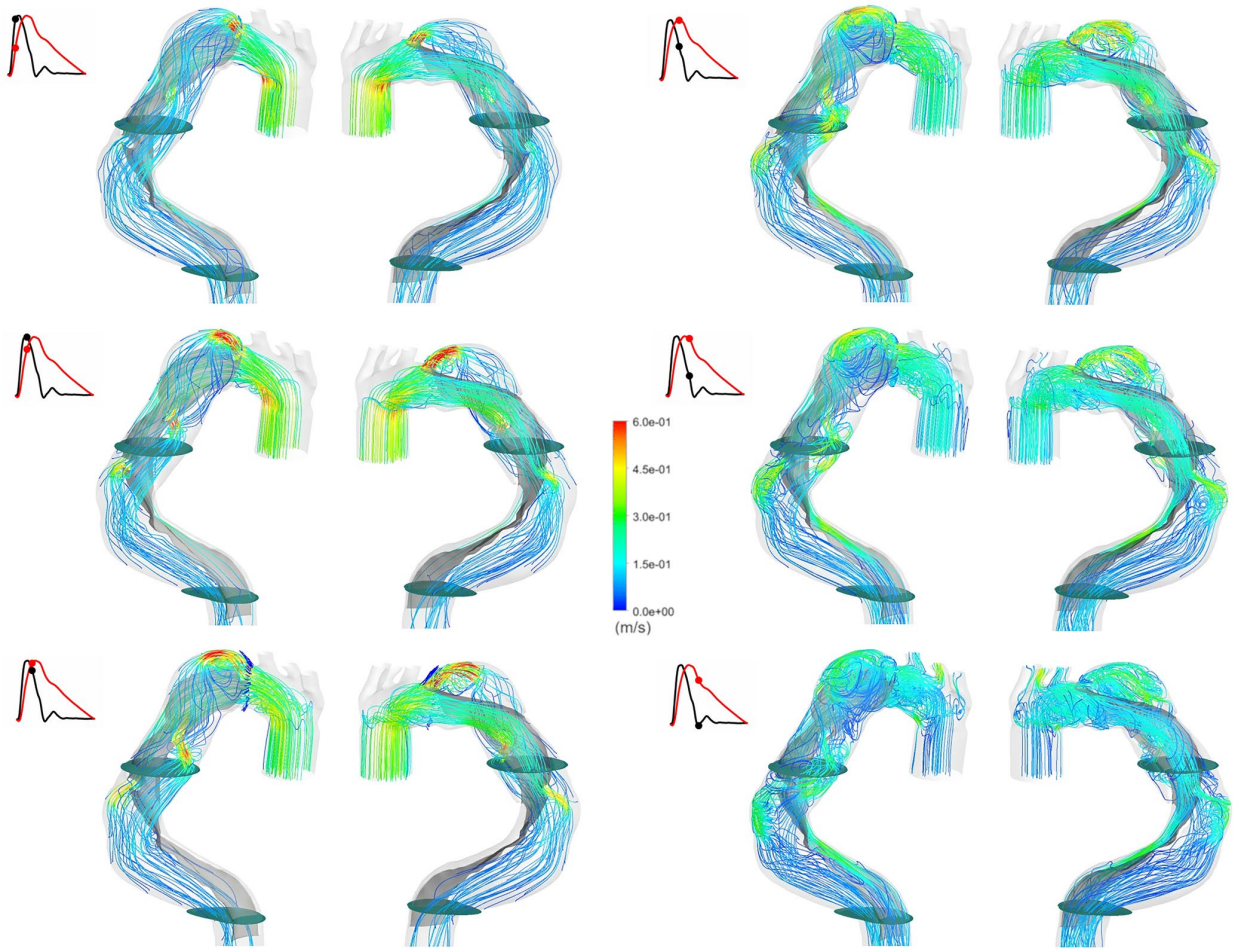
Streamlines for F06W12 at different instants over the cardiac cycle. Black and red curves are respectively flow rate profile and pressure profile in function of time.

In a general way and whatever the case, the flow hemodynamic is driven by the complex geometry including here several curvatures, the presence of narrowing: an entry and 2 re‐entry tears along the flap length and the occurrence of enlargement in FL. Figure 2 shows the instantaneous velocity streamlines for 3 instants in the cardiac cycle for F06W12. In detail, the main features are a jet formation through ET, RET1 and RET2 during the systolic phase, entering the false lumen and tending to impact the opposite wall. As the flow rate decelerates, the flow patterns become more complex and jets are surrounded by recirculation areas in the widening part of FL. During the diastolic phase, flow patterns are increasingly disturbed and progressively reach zero values by the end of the cardiac cycle.

**FIGURE 3:**
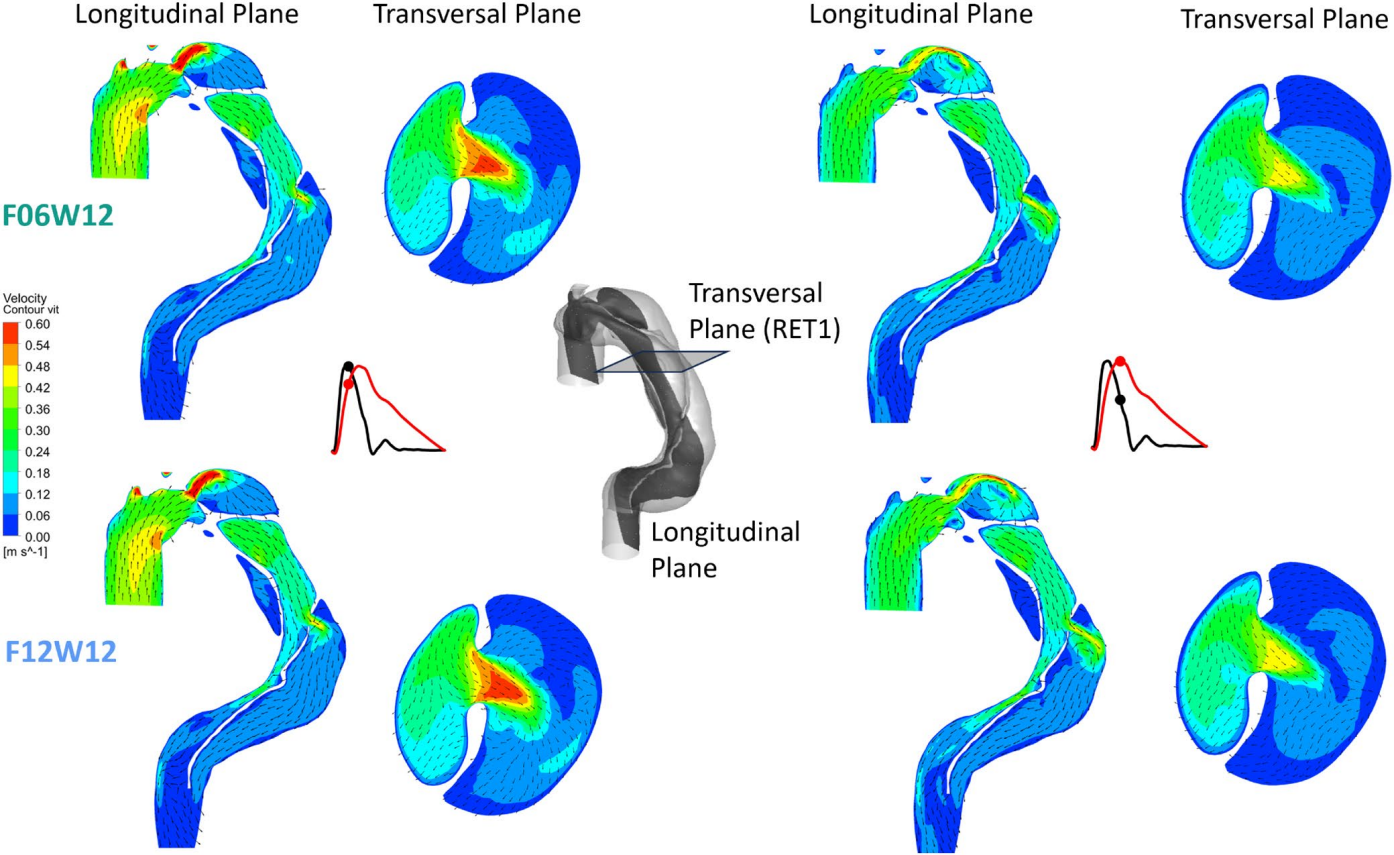
Velocity patterns in two perpendicular planes (Transversal and Longitudinal Planes) for F06W12 (upper line) and F12W12 (lower line) at 2 different instants (peak of flow rate, on the left; peak of pressure on the right). Black and red curves are, respectively, flow rate profile and pressure profile in function of time.

As the structural displacements in F06W12 and F12W12 are equivalent, the overall flow features are also equivalent as can be observed on the velocity patterns and vectors shown in Figure 3. Therefore, we focus the analysis mainly on the flow patterns in F06W12 and F06W27.

#### 3.2.1 Flow Rate

**FIGURE 4:**
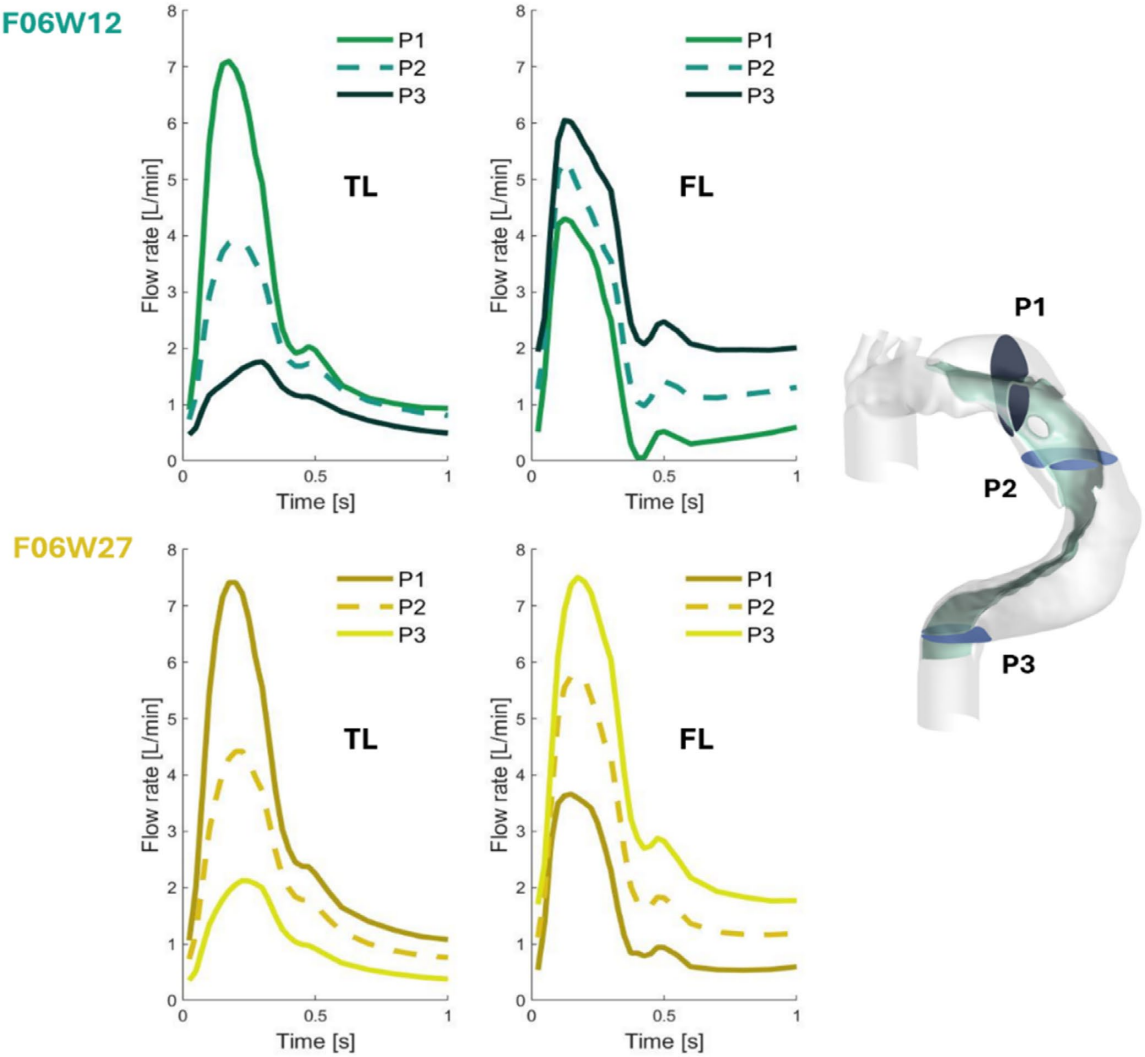
Flow rate in true lumen (TL) and false lumen (FL) over a cardiac cycle, for F06W12 (upper line) and F06W27 (lower line). Three planes P1, P2, P3 are investigated; their localisation is given on the right: P1 is between the first entry tear (ET) and the first re‐entry tear (RET1); P2 is located between the first and the second re‐entry tear. P3 is located between the second re‐entry tear and the outlet.

To quantify the flow rates in both true and false lumens three planes were defined: P1 after ET, P2 between RET1 and RET2, and P3 after RET2 (Figure 4). Table 3 shows the average flow rate over a cardiac cycle in TL and FL through the three planes and expressed as a percentage of the total inlet flow rate. The peak flow rate in TL is observed at *t* = 0.175 s with a value of 7.10 L/min and at *t* = 0.175 s with a value of 7.42 L/min for F06W12 and F06W27 respectively. In P2, the flow rate in both lumens tends towards equilibrium. The peak flow rate occurs in TL later than in FL in whatever case, with a value of 3.91 L/min for F06W12 and 4.42 L/min for F06W27. In P3, the mean flow rate in TL is smaller than in FL in both cases. The peak flow rate in TL is observed at *t* = 0.3 s with a value of 1.76 L/min for F06W12 and at *t* = 0.225 s with a value of 2.13 L/min for F06W27.

**TABLE 3:** Average flow rate over a cardiac cycle in TL and FL expressed as a percentage of total inlet flow rate, for each plane P1, P2 and P3 and each configuration.

	F06W12			F06W27		
	P1	P2	P3	P1	P2	P3
TL	71.3%	47.9%	25.6%	69.2%	45.0%	74.4%
FL	28.7%	52.1%	22.5%	30.8%	55.0%	77.5%

FL is therefore driven by the flow coming from the TL along the entire length of the flap, with a lower flow than that of the TL at the beginning (P1) and a higher flow at the end (P3). We notice that the softer the wall, the earlier the peak flow appears in FL.

#### 3.2.2 Pressure Difference

**FIGURE 5:**
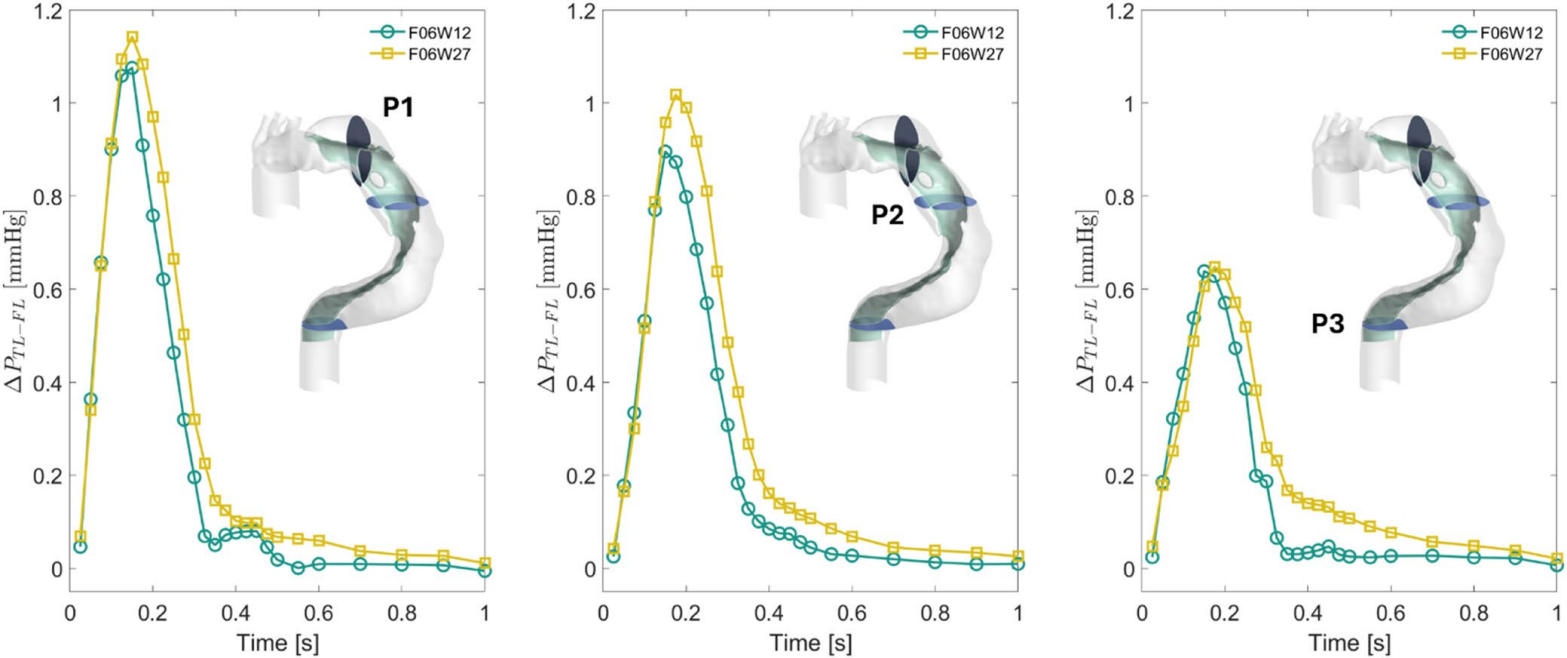
Evolution of the pressure difference between true lumen (TL) and false lumen (FL) over a cardiac cycle for P1 (on the left), P2 in the middle, and P3 on the right. F06W12 results are green line with circle marker; F12W12 are yellow line with square marker.

Whatever the case, the instant in the cardiac cycle and, the plane considered, the difference between the mean pressure recorded in the TL and the FL (Δ*P*_TL‐FL_) is always smaller than 1.2 mmHg (Table 4). In plane P1, respectively P2 and P3, Δ*P*_TL‐FL_ for F06W12 is higher than for F06W27 up to *t* = 0.1 s, *t* = 0.125 s and *t* = 0.175 s, respectively (Figure 5). Then, Δ*P*_TL‐FL_ in F06W12 is always lower than in F06W27. The Δ*P*_TL‐FL_ peak value is reached at *t* = 0.150 s for F06W12 in all the planes, whereas for F06W27, it is slightly temporally shifted from *t* = 0.150 s in P1 to *t* = 0.175 ms in P2 and P3. The Δ*P*_TL‐FL_ peak value is equal to 1.143 mmHg in P1 for F06W27, respectively, 1.076 mmHg for F06W12. In P2, the Δ*P*_TL‐FL_ peak value is 13.7% higher for F06W27 than for F06W12. In P3, the Δ*P*_TL‐FL_ peak value is 1.45% higher for F06W27 than for F06W12.

**TABLE 4:** Mean and max Δ*P*_TL‐FL_ over a cardiac cycle, for cases F06W12 and F06W27 and for all investigated planes.

	F06W12			F06W27		
	P1	P2	P3	P1	P2	P3
Mean Δ*P*_TL‐FL_ (mmHg)	0.30	0.28	0.19	0.38	0.36	0.25
Max Δ*P*_TL‐FL_ (mmHg)	1.08	0.90	0.64	1.14	1.02	0.65

## 4 Discussion

Three mechanical behaviour cases are investigated in the same RTAD geometry to understand and highlight the main role and influence of the flap and wall independently. All cases exhibit similar trends as far as flow patterns, structural displacements and stresses are concerned but the intensities are different. Even if F06W12 and F12W12 seem very close in terms of overall mechanical behaviour, values are quite different in F06W27, tending to prove that the wall is the main driver of the whole mechanical behaviour of RTAD. We indeed show that a stiffer wall reduces flap motion by half when comparing F06W12 with F06W27. The maximum displacements that are observed in this study are found for the F06W12 case and are between 2 and 3 mm.

The question of the flap displacement values is often raised in the literature on TAD FSI models and discussed in relation to the values found in imaging studies. In FSI models [5, 21] where Young's modulus of the wall is approximately the same as that of the flap (ratio (*E*_*w*_/*E*_*f*_) ϵ [0.83; 1.53]), flap displacement is often found to be about 1–2 mm. On the contrary, values of flap displacement up to more than 10 mm have been reported in imaging studies [22]. For example, Baumler et al. [4] had to drastically reduce the flap Young's modulus down to 20 kPa, to capture such a flap displacement value in a FSI model. With a wall Young's modulus of 800 kPa in the latter study, the ratio (*E*_*w*_/*E*_*f*_) was equal to 40. It is important to underline that the authors had to reduce the Young's modulus of the flap to values that are very low in physiological terms and even lower in pathological terms. It is therefore interesting to focus on the natural history of the pathology to address these a priori diametrically opposed ranges of values. Traditionally, 3 main temporal phases are used to classify the TAD: the acute phase which lasts for the first 2 weeks after the onset of symptoms followed by a subacute phase lasting from 2 weeks to 1 month, and finally the chronic phase after 1 month [23]. The transition from acute to chronic phase is of interest as some changes in the pathological description may occur during this period. In a retrospective study dedicated to this topic, Peterss et al. [24] observed a rapid decrease in the amplitude of flap displacement over time during the transition, as well as flap thickening, with an exponential variation. Indeed, from a clinical point of view, a flap with little movement is generally considered to be an indicator of the chronic status of the TAD. For instance, Orabi et al. [25] showed that patients in the acute phase present a flap mobility of 6.62 mm and a flap thickness of 2.90 mm whereas those in the chronic phase present a flap mobility reduced to 1.69 mm and thickness increased up to 4.01 mm. Similar results are found in Murayama et al. [26] who defined a ‘pulsating’ and a ‘static’ type of flap motion that are shown to be generally linked to the acute and chronic status respectively. In addition, concerning the aortic wall, it was shown through mechanical characterisation in chronic TAD patients that a general stiffening is observed for all patients in comparison to healthy ones [27]. Global structural motion is thus reduced in TAD chronic phase, suggesting a rapid remodelling of flap and wall during the acute phase leading to mechanical changes. A review [24] highlighted that over 90% of TAD aortic tissue specimens demonstrated significant fibrosis and lamellar elastin fragmentation within a few months; this transition coincides with the apparent thickening and loss of mobility of the dissected flap noted on imaging studies of chronic dissections [28].

If we correlate these clinical observations on flap mobility with the present numerical results, it appears that one reason for the decrease in flap mobility could be explained by an increase in wall stiffness during the chronic phase: flap mobility is significantly reduced in F06W27 compared with F06W12. It appears that the flap movement is reduced, even more so in pathological walls which are known to be stiffer [27]. Choosing a behaviour that is too compliant for the wall and/or the flap does not seem relevant in a modelling case aimed at helping the clinical question, knowing that RTAD patients are more prone to reintervention in the chronic phase than in the acute phase. However, proposing either only a compliant flap but a rigid wall or entirely rigid models also fails to capture the specificities of the flow and over‐ or underestimates biomechanical quantities [7]. It is therefore essential to model the right mechanical behaviour of the wall and the flap.

The main clinical question that FSI models can address is the anticipation of the evolution of the pathology and its ultimate consequence, that is, the adverse event (from aortic dilatation beyond the 50 mm threshold up to rupture). Adverse events evolve over a different time frame for each patient with RTAD, and the dissections that remain ‘uncomplicated’ for a period of time may present a clinical dilemma in deciding if and when a preventive re‐intervention to place an endovascular prosthesis is required [29, 30]. Thus, outside the acute phase, the flap motion is low, although present, and the focus should be on correctly quantifying the resistance to flow in both lumens, which in turn could lead to wall distension and diameter increase.

In the present study, we show that the pressure difference between both lumens remains low in all cases. Wall and flap behaviour thus have a very minor influence here. However, it is important to highlight the presence of 2 re‐entries along the length of the flap, which should allow a pressure equilibrium between the false and true lumens that does not exceed a few mmHg. Zimmermann et al. [2] showed that a modification of entry or exit tear area can modify the value of the difference of pressure between the false and true lumen, including its sign. It is worth noting that in their study, there was no re‐entry along the dissection. The in vitro study by Tsai et al. [31] confirmed that a dissection with 2 tears leads to a lower pressure difference between false and true lumens (< 1 mmHg on average) than a single tear. Clinically, the number of tears has also been found to influence aortic growth in patients: in patients with only one tear it may be the cause of higher growth rates [32]. A second point related to flow resistance is the presence of a patent lumen. The patency of the false lumen is the other main criterion controlled by the clinician to follow patient evolution. In this sense, the integration of a thrombus model including its current permeability in future FSI models may be of interest.

Some limitations must be mentioned, such as the choice of boundary flow conditions that were not patient specific, or the choice of the wall and flap mechanical behaviour that were simplified in regard to reality. However, the aim of the present study was to compare 3 cases and not to obtain an absolute quantification of biomechanical quantities. We can also mention the need to embed the aortic root using the FSI model, whereas the ascending aorta is known to be mobile due to its proximity to the heart [33]. However, the presence of the relatively rigid stent graft in the case of RTAD strongly constrains movement making this working hypothesis acceptable.

## 5 Conclusion

This study emphasises the importance of modelling both the wall and the flap to capture specific structural mechanical patterns. Wall stiffening is a key consideration in FSI modelling, as our findings demonstrate its role in driving overall structural motion in (R)TAD. This can be particularly important when trying to quantify stresses and strains in the context of wall rupture for instance.

But, we have also demonstrated that a stiffer pathological wall leads to smaller flap displacements, which is consistent with clinical observations in the chronic phase. Therefore, to gain new insights into the prediction of clinical evolution of dissection, it is advisable to quantify the resistance to flow and permeability of the false lumen by analysing flows rather than focusing on the mechanical behaviour of the flap and wall. In this context, one computational alternative is to define the movements of the wall and flap as boundary conditions of a fluid domain, to account for even slight movements while avoiding FSI studies that incorporate non‐patient‐specific global behaviour laws. This approach would provide insight into the evolution of pathology while minimizing computation time.

## Ethics Statement

Approval number 2019‐48.

## Conflicts of Interest

The authors declare no conflicts of interest.

## Data Availability

The data that support the findings of this study are available from the corresponding author upon reasonable request.
